# Asymptomatic Bacteriuria in Patients with Type 2 Diabetes Mellitus

**DOI:** 10.3390/idr15010005

**Published:** 2023-01-05

**Authors:** Georgia Matthiopoulou, Petros Ioannou, Anna Mathioudaki, John A. Papadakis, Vasiliki N. Daraki, Angelos Pappas, Sotiris Souris, Sofia Maraki, Chrysoula Stathopoulou, Diamantis P. Kofteridis

**Affiliations:** 1 First Department of Internal Medicine, Venezeleion General Hospital of Heraklion, 714 09 Heraklion, Greece; 2 School of Medicine, University of Crete, 700 13 Heraklion, Greece; 3 Department of Internal Medicine and Infectious Diseases, University Hospital of Heraklion, 711 10 Heraklion, Greece; 4 Department of Endocrinology, University Hospital of Heraklion, 711 10 Heraklion, Greece; 5 Diabetes Unit, Venezeleion General Hospital of Heraklion, 714 09 Heraklion, Greece; 6 Department of Microbiology, University Hospital of Heraklion, 711 10 Heraklion, Greece; 7 Laboratory of Rheumatology, Autoimmunity and Inflammation, Institute of Molecular Biology and Biotechnology, 700 13 Heraklion, Greece

**Keywords:** diabetes, urinary tract infection, asymptomatic bacteriuria, urine culture, vitamin B12

## Abstract

Objectives: Asymptomatic bacteriuria (ASB) is a common finding in patients with diabetes. Moreover, patients with diabetes and ASB have a greater risk for symptomatic urinary tract infections and associated severe complications. The aim of this study was to estimate the prevalence of ASB, as well as to identify independent risk factors and related pathogens associated with ASB in female and male patients with type 2 diabetes mellitus (T2D). Methods: This prospective case-control study was performed at the University hospital, and the Venezeleion General Hospital, Heraklion, Greece between 2012 and 2019. All patients with T2D attending the diabetes and hypertension outpatient clinics at both hospitals were enrolled, and data regarding their medical history and clinical and laboratory profiles were recorded. Asymptomatic patients with positive urine cultures were assigned as cases while those with negative urine cultures were designated as controls. Results: A total of 437 adult patients of which 61% were female and 39% were male patients with a mean age of 70.5 ± 9.6 years, were enrolled. The prevalence of ASB was 20.1%, in total. ASB was noted in 27% of female participants and 9.4% of male participants. Higher glycated hemoglobin (OR = 3.921, 95%CI: 1.521–10.109, *p* < 0.001) and urinary tract infection within the previous year (OR = 13.254, 95%CI: 2.245–78.241, *p* < 0.001) were independently positively associated with ASB, while higher levels of vitamin B12 were independently negatively associated with ASB (OR = 0.994 per ng/mL, 95%CI: 0.989–0.999, *p* < 0.001). Conclusions: Development of ASB was associated with specific factors, some of which may be modifiable. Interestingly, high B12 was found to be negatively associated with ASB.

## 1. Introduction

Diabetes mellitus (DM) is the most common endocrinological disease, occurring with increasing prevalence, which has risen worldwide from 108 million cases in 1980 to 537 million cases in 2021 [1]. According to the International Diabetes Federation, the total number of people living with diabetes is predicted to rise to 643 million by 2030 and 783 million by 2045 [1].

Asymptomatic bacteriuria (ASB) is a common finding in many populations, including patients with DM, pregnant women, the elderly, and patients with impaired voiding [2,3,4]. The majority of studies suggest a three-fold higher prevalence of ASB in patients with DM [5] and there is also evidence that ASB confers an increased risk of symptomatic urinary tract infections (UTIs) in this specific population [5,6,7].

Several risk factors, including age, sexual intercourse, as well as duration, metabolic control, and complications of diabetes have been associated with the presence of ASB in patients with DM thus far [8,9,10]. Even though there are references reporting a correlation between impairment of metabolic control and increased risk of urinary tract infection (UTI) [11,12], in patients with DM, the association between glycated hemoglobin (HbA1c) levels and ASB is not yet well explained [6,7]. Moreover, the association between advanced age and ASB in patients with DM remains questionable [11,13,14,15]. There is evidence that longer duration of diabetes is correlated with ASB [6,10,11,13], although this association was not confirmed in other studies [9,16,17]. Furthermore, body mass index (BMI), sexual intercourse, a prior history of UTI, in addition to pyuria and diabetes complications, have also been reported as risk factors for ASB in patients with DM, even though related studies are conflicting [11,17,18].

Determination of preventable risk factors related to ASB in patients with DM is deemed important, since early recognition and management of these factors could prevent the presence of ASB in this specific group of patients, thus improving their quality of life and reducing associated health care costs. In addition, considering that ASB may lead to symptomatic and severe UTI [5,6,7], identification and elimination of related predisposing factors may minimize complications and may also facilitate the diabetic patient’s management. However, treatment of ASB in patients with diabetes is not recommended, except in pregnant women or prior to urological procedures [19].

The aim of this study was to estimate the prevalence of ASB and identify risk factors and causative microorganisms relating to ASB in men and women with type 2 diabetes mellitus (T2D).

## 2. Materials and Methods

### 2.1. Study Population and Enrollment

Six hundred and forty-four patients aged more than 18 years, with a diagnosis of T2D, according to the American Diabetes Association diabetic definition standard [20], were enrolled over a seven-year period (from 2012 to 2019) from the diabetes and the hypertension outpatient clinics of the University Hospital and the Venezeleion General Hospital of Heraklion, Crete, Greece. Exclusion criteria were: type 1 diabetes; recent hospitalization, or surgery (within the past six months before presentation); use of anti-microbials either prophylactic or therapeutic (within the previous two weeks); symptomatic UTI at the time of evaluation (e.g., symptoms of dysuria, fever, frequent urination, lower abdominal or flank pain); use of corticosteroids or other immunosuppressive agents; known anatomical or functional abnormality of the urinary tract; presence of lithiasis; pregnancy; chronic prostatitis and recent urinary tract instrumentation (within the past one month before presentation) or presence of an indwelling urine catheter, or a nephrostomy tube.

All patients with T2D of the final study population gave informed written consent.

### 2.2. Study Design and Methods

Patients with T2D presenting to the outpatient diabetic and hypertension clinics were prospectively enrolled and evaluated. Patients who had a positive diagnosis of ASB i.e., women with two consecutive urine cultures with the presence of the same microorganism in quantitative counts of ≥10^5^ cfu/mL and men with a single urine culture with the presence of a bacterium at quantitative counts of ≥10^5^ cfu/mL, were enlisted as cases. Patients with negative urine cultures were enlisted as controls.

A complete history was obtained from all patients at their first visit with a standard questionnaire. Data regarding age, gender, family history of diabetes, duration of diabetes, medical history, current medications and complications of diabetes, recent sexual intercourse (within one week), previous antimicrobial use (within three months) or UTI within the last twelve months and a history of recurrent UTIs were recorded. Body weight, height, BMI, and findings from physical examination including systolic and diastolic blood pressure, heart rate and temperature, as well as respiratory, cardiovascular, and abdominal examination, were also recorded upon first visit. In addition, laboratory data relating to urine analysis, urine culture and blood analysis data were evaluated. Additional information was retrieved from patient’s medical records.

Blood examinations were ordered at the discretion of the treating physicians at the outpatient clinic and were collected upon patient’s first presentation. Metabolic control was estimated by the determination of HbA1c upon patients’ first attendance. Peripheral blood sampling was performed for the evaluation of HbA1c and vitamin B12 levels. HbA1c levels were measured using the Menarini HA-8180 Arkray high performance liquid chromatography (HPLC) analyzer, Menarini Industrie Farmaceutiche Riunite S.r.I. The Alinity i system (Abbott Laboratories, Chicago, IL, USA) chemiluminescent microparticle immunoassay (CMIA) analyzer was used for the evaluation of serum vitamin B12 levels.

The study was approved by the Ethic Committee of the Venezeleion General Hospital and the University Hospital of Heraklion, Crete, Greece.

### 2.3. Urine Cultures

All study participants were consulted for proper urine specimen collection in order to avoid contamination, and a clean urine specimen was collected in appropriate sterile box containers from all patients on first visit. All women with an initial positive urine culture gave a second urine culture within a week, according to the 2005 Infectious Diseases Society of America (IDSA) ASB guidelines [21]. They were also advised to avoid sexual contact during that one-week period. Urine samples were immediately refrigerated at 4 °C and were available for laboratory analysis within one hour of collection.

Urine analysis was carried out according to standard procedures and urine cultures were performed in positive cases by standard techniques using Columbia blood and MacConkey agar plates (BioMérieux, Marcy l’ Etoile, France). Bacterial species were identified by the use of standard biochemical methods and the Vitek 2 automated system (BioMérieux SA, Marcy-l'Étoile, France).

### 2.4. Definitions

ASB was defined as the presence of a freshly voided midstream urine specimen that yields positive cultures, with at least 10^5^ colony forming units (cfu)/mL, of the same bacterium in a patient that does not have symptoms indicative of a UTI. Identification of the same microorganism in two consecutive cultures was necessary to confirm the diagnosis of ASB in the female participants of the study, while in male participants one positive culture was deemed adequate [21].

Patients with ≥3 episodes of uncomplicated UTIs within the last 12 months before enrollment or ≥2 episodes of uncomplicated UTIs within the last 6 months before presentation were considered as having recurrent UTIs [22]. Recent sexual intercourse was defined as sexual intercourse within the last week before the patient’s enrollment. Albuminuria was defined either as a spot urine albumin to creatinine ratio of 30–300 mg/g or >300 mg/g [23].

A contaminated urine sample was defined as a sample with the presence of 2 or more different microorganisms in the same urine specimen, each with a quantitative count of less than 10^5^ cfu/mL. Patients with contaminated urine samples were excluded from the study.

### 2.5. Statistics

Categorical data were analyzed with Fisher’s exact test. Continuous variables were compared using Student’s *t*-test for normally distributed variables and the Mann–Whitney U-test for non-normally distributed variables. All tests were two-tailed and *p*-values < 0.05 were considered to be significant. Data are presented as number (%) for categorical variables and median (interquartile range, IQR) or mean [±standard deviation, (SD)] for continuous variables. A univariate logistic regression analysis model was developed to evaluate the effect of gender, age, BMI, duration of diabetes, HbA1c, medical history and findings from physical examination, prior antimicrobial use, prior UTI, recurrent UTIs, recent sexual intercourse, laboratory parameters such as white blood cell count, hemoglobin and hematocrit levels, platelet count, erythrocyte sedimentation rate, C-reactive protein levels, plasma glucose concentration, vitamin B12, urea and creatinine serum levels, as well as findings from the urine analysis, on developing ASB. GraphPad Prism 6.0 (GraphPad Software, Inc., San Diego, CA, USA) was used to calculate related statistics for all of the above. A multivariate logistic regression analysis model was developed to evaluate the association of factors identified in the univariate analysis with a *p* < 0.05. Multivariate analysis was performed using the SPSS version 23.0 (IBM Corp., Armonk, NY, USA).

## 3. Results

**Figure 1:**
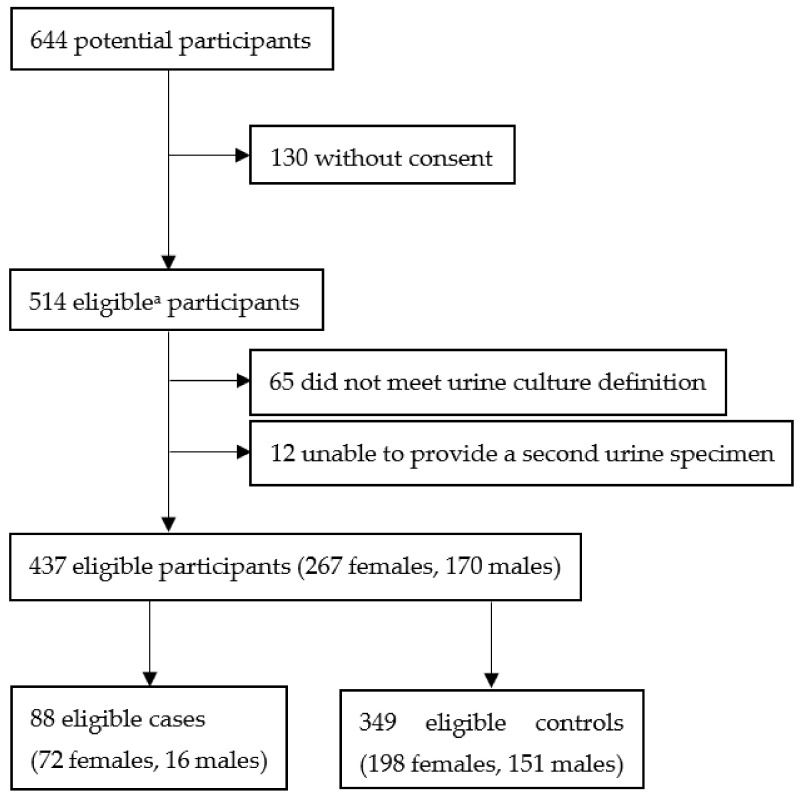
Flowchart of the study population. ^a^ Eligibility criteria described in the text.

As shown in the flowchart of the study population (Figure 1), 514 of the 644 participants gave informed written consent (response rate 79.8%). The final eligible study group consisted of 437 patients [267 female (61%) and 170 (39%) male patients, mean age 70.5 ± 9.6 years] after the exclusion of 48 patients with urine cultures yielding two or more different microorganisms (in a quantitative count of less than 10^5^ cfu/mL) and 12 female patients who did not provide a second urine culture, as well as 17 female patients with a negative second urine culture (Figure 1). Table 1 presents the baseline characteristics of the study population.

Antimicrobial use within the last 3 months was noted in 6.6%, while 17.8% of all patients had experienced a UTI in the last year before evaluation. Additionally, 2.7% had experienced recurrent UTIs, and 6.8% had a history of recent sexual intercourse. In 27% female participants and in 9.4% male participants ASB was noted. In total 20.1% presented ASB.

The characteristics of patients with T2D with and without ASB are shown in Table 2. Several statistically significant differences were noted, as, for example, in terms of age, BMI, duration of T2D, mean HbA1c, prior antimicrobial use, prior UTI, recurrent UTIs, recent sexual contact, serum B12 levels, albuminuria, nitrites and leukocyte esterase in the urine. More specifically, the characteristics of T2D female patients -with and without ASB- are shown in Table S1. Several factors were correlated with ASB in female patients with T2D, and more specifically, age, duration of diabetes, BMI, HbA1c, recurrent UTIs, sexual intercourse, albuminuria, presence of leucocyte esterase or nitrites in the urine analysis, recent use of antimicrobials (in the previous three months) and a history of UTI in the last 12 months. Moreover, women with T2D and ASB exhibited lower serum levels of vitamin B12 compared to women with T2D but without ASB. Differences in clinical and laboratory parameters were noted between male patients with T2D and ASB and male patients with T2D but without ASB, as shown in Table S2. Male patients with T2D and ASB had a higher HbA1c and BMI, recurrent UTIs, a history of UTI, prior antimicrobial use and albuminuria, nitrites, and leukocyte esterase in their urine. No statistically significant relation was noted between findings from physical examination, complications of diabetes and ASB in men and women with T2D.

The results of a comparison between male and female patients with T2D and ASB, showed that only BMI (*p* = 0.0455) and family history of diabetes (*p* = 0.0468) were significantly different between the two groups (Table 3). Female patients with T2D and ASB had a slightly higher BMI compared to male patients with T2D and ASB (33.50 ± 0.5992 vs. 30.73 ± 1.050) and a more frequent family history of T2D (*p* = 0.0468).

*Escherichia coli* was the most frequently isolated microorganism which was identified in more than half of all cases (62.5%). *Streptococcus agalactiae* was identified in 9%, *Klebsiella pneumonia*, *Enterococcus faecalis* and *Proteus mirabilis* in 6%, *Pseudomonas aeruginosa* in 2%, and *Staphylococcus haemolyticus*, *Acinetobacter lwoffi*, *Providencia stuartii*, and *Streptococcus bovis* in 1% of the cases (Table 4).

The most commonly isolated microorganisms were gram-negative bacteria, which were identified in 80.7% of all cultures obtained from patients with T2D and ASB. Male patients with T2D and ASB had less gram-negative isolates (62.5% vs. 84.7%, *p* = 0.0736) than female with T2D and ASB even though this result did not reach statistical significance.

Among all available clinical and laboratory parameters that were included in the logistic regression analysis, only HbA1c, vitamin B12 and a history of UTI were found to be independently associated with the development of ASB. Table 5 shows the results of the logistic regression analysis, and more specifically, all parameters that were found to be associated with the development of ASB in the univariate and in the multivariate logistic regression analysis.

Higher levels of HbA1c (OR = 3.921, 95%CI: 1.521–10.109, *p* < 0.001) and a UTI episode within the last 12 months (OR = 13.254, 95%CI: 2.245–78.241, *p* < 0.001), were found to be independently, positively associated with the development of ASB in the multivariate logistic regression analysis. In the same analysis, higher levels of vitamin B12 were found to be independently, negatively associated with the development of ASB in patients with T2D (OR = 0.994 per ng/mL, 95%CI: 0.989–0.999, *p* < 0.001).

## 4. Discussion

The present study has shown that the prevalence of ASB among patients with T2D was 20.1%. Presence of ASB showed a positive correlation with higher HbA1c levels and recent UTI, while higher B12 levels were found to be negatively associated with the occurrence of ASB in patients with T2D.

Diabetes has been recognized as a condition associated with higher rates of ASB, even though this association has been documented in women but remains unconfirmed in men [8,24]. This predisposition could be explained by differences in host responses between patients with and without diabetes, or by differences regarding the invading microorganisms.

In general, studies referring to the association between diabetes and ASB as well as the prevalence of ASB in patients with DM, are relatively old [8,9,13]. These studies reported a 9–27% prevalence of ASB in women with DM and 0.7–11% in men with DM [8]. In the present study, 27% female participants and 9.4% male participants with T2D developed ASB and an overall prevalence of 20.1% was noted. In a study by a group from Turkey with 123 patients with T2D, 22 patients developed ASB, a finding similar to the one found in the present study [13]. In a more recently published study by Zaidi et al. which comprised 667 women with T2D, a prevalence of ASB equal to 19% was reported, which is also a finding similar to our study [11]. By contrast, a case control study published by a group from Israel, in which 411 women with T2D were enrolled, reported a 6.1% prevalence of ASB [16].

In the aforementioned study from Turkey, female gender, HbA1c levels, duration of diabetes and pyuria were identified as risk factors for ASB in patients with DM [13]. Herein, we also found higher HbA1c, longer duration of diabetes, female predominance, as well as more frequent presence of urine leucocyte esterase in patients with ASB and T2D, compared to patients with T2D but without ASB.

However, in the present study, several other statistically significant differences were identified between T2D patients with and without ASB, and there has also been an attempt to distinguish between male and females’ risk factors related to ASB. Moreover, a regression analysis was performed aiming to identify independent factors associated with the occurrence of ASB in patients with T2D.

In the study by Zaidi et al., recent antimicrobial use, a history of UTI, higher levels of HbA1c and a longer duration of diabetes were associated with the presence of ASB in patients with DM [11]. These conditions were also identified as risk factors in the multivariate analysis of the present study, although the frequent presence of proteinuria in patients with T2D and ASB was not confirmed in our multivariate analysis.

Notably, Ishay et al. found no relation between ASB and HbA1c levels, age, BMI, and duration of diabetes but only identified albuminuria and serum creatinine to differ between diabetic women with and without ASB [16]. It is worth noting that, women included in the study and control groups of the aforementioned study were recruited from a single hospital and the definition of ASB was based on a single adequate positive urine specimen and not on two consecutive specimens, as in our study. Moreover, the study included only Jewish and Muslim women with a religious and cultural education that may influence their sexual behaviour and consequently the frequency and the risk factors of bacteriuria.

Reports referring to the risk factors related to ASB in men with DM are lacking. The present study has shown that higher HbA1c and higher BMI, prior antimicrobial use, a history of UTI within the last year, a history of recurrent UTIs and the presence of albuminuria, nitrites and leukocyte esterase in the urine analysis, may be considered as risk factors for ASB in men with T2D.

To our current knowledge, there are no studies regarding the potential differences between male and female patients with DM and ASB. In a sub-analysis of the present study, it has been documented for the first time that female patients with T2D and ASB have a higher BMI and a more frequent family history of diabetes, than male patients with T2D and ASB.

Regarding the microbiology of ASB, gram-negative bacteria were identified in 80.7% of the urine cultures obtained from patients with T2D and ASB. Additionally, gram-negative pathogens were more common in female patients with T2D and ASB than in male patients with T2D and ASB (84.7% vs. 62.5%). A prevalence of *E. coli* comparable to the one found in this study (62.5%) has been reported in some studies [13,25]. *Klebsiella pneumoniae* has been referred as the second most frequent pathogen in the urine cultures of diabetic patients with ASB, while, herein, *Streptococcus agalactiae* was the second most frequent pathogen in diabetic patients with ASB [13,25]. Microbiology of ASB identified in the present study is in accordance with that reported in previous studies [25,26].

Interestingly, using a multivariate logistic regression analysis model, high serum levels of vitamin B12 were identified to be negatively associated with the occurrence of ASB in patients with T2D. There has been no evidence so far in the literature suggesting a protective role of vitamin B12 in developing ASB. However, vitamin B12 is known to be involved in intestinal immune regulation, since gut microbes use it as a cofactor for metabolic pathways, thus supporting the intestinal immune barrier [27,28,29]. In addition, vitamin B12 has an immunomodulatory role in cellular immunity as it has been described in animal models where macrophages from B12-deficient mice exhibited increased levels of pro-inflammatory TNF-α, while lower levels of IL-6 were identified in B12-deficient rats. These conditions were reversed after administration of sufficient doses of vitamin B12 in both animal models [30]. Notably, lower urine levels of IL-6 in children have been associated with the presence of ASB [31]. Furthermore, vitamin B12-deficient patients with anemia, have lower numbers of all lymphocytes, a change in Th/Tc cell ratio, and suppressed NK cell activity [30]. Considering that host resistance to UTI relies strongly on innate immunity along with the potential immunomodulatory role of the vitamin B12, it could be proposed that lower levels of this vitamin influence the innate immune response in the urinary tract system favoring an asymptomatic carriage status. However, since gut dysbiosis is a well-recognized entity that may affect the interplay of absorbed metabolites that is commonly seen in patients with DM, the finding of reduced vitamin B12 could, theoretically, be an epiphenomenon seen in more deranged patients with DM that happen to develop ASB more often [32]. In that case, restoration of B12 levels would not have any effect on the development of ASB. Thus, more studies are needed to that direction, in order to clarify the interplay between vitamin B12 and development of ASB, a prodrome of UTI. If, however, higher levels of vitamin B12 could prevent the establishment of ASB in patients with DM, design of future studies investigating the role between vitamin B12 and development of UTIs would also be reasonable.

This study has some limitations that should be noted. First, the study sample is relatively small; therefore, the results should be read cautiously. Furthermore, in terms of age all adult patients were eligible to be enrolled, mean age was high. Thus, the present data may be more applicable to older patients. In addition, this is a study which was conducted in only two hospitals in the same city, so further studies should be performed in order to validate these results in larger populations. Moreover, parameters studied herein are those usually studied in an outpatient clinic, selected at the discretion of the treating physician, thus, associations with other factors, not assessed in this study, could have been missed. Finally, some parameters, such as whether the patients of the present study were on B12 supplementation, was not recorded.

## 5. Conclusions

The present study has shown that the overall prevalence of ASB among patients with T2D was 20.1%. The prevalence of ASB among male and female patients with T2D was 9.4% and 27%, respectively. *E. coli* was the most common organism causing ASB in both male and female participants in our study. In women with T2D, age, duration of diabetes, BMI, HbA1c and recurrent UTIs have been identified as risk factors for ASB, while in men with T2D, HbA1c, BMI, recurrent UTIs, previous UTI, recent antimicrobial use and the presence of albuminuria, nitrites and leukocyte esterase in urine were correlated with ASB. Using a multivariate logistic regression analysis, the presence of ASB showed a positive correlation with higher HbA1c levels and an episode of UTI within the last year, while most significantly, higher B12 levels were found to be negatively associated with development of ASB in patients with T2D.

## Figures and Tables

**Table 1:** Baseline characteristics of the study population.

Characteristics	All Patients (*n* = 437)	Women with T2D (*n* = 267)	Men with T2D (*n* = 170)
ASB, *n* (%)	88 (20.1%)	72 (27%)	16 (9.4%)
Age (years), mean (SD)	70.4 (9.6)	70.6 (9.7)	70 (9.6)
BMI (kg/m^2^), mean (SD)	30.2 (5.8)	31.5 (6)	28.1 (4.8)
Duration of DM (years), median (IQR)	12 (7–18.3)	12 (7–19)	12 (7–16)
Family history of DM, *n* (%)	278 (63.6)	176 (65.9)	102 (60)
HbA1c, %, mean (SD)	6.8 (0.9)	6.9 (0.9)	6.8 (1)
Prior antimicrobial use, *n* (%)	29 (6.6)	15 (5.6)	14 (8.2)
UTI within one year, *n* (%)	78 (17.8)	68 (25.5)	10 (5.9)
Recurrent UTIs, *n* (%)	12 (2.7)	9 (3.4)	3 (1.8)
Recent sexual contact, *n* (%)	30 (6.9)	18 (6.7)	12 (7.1)
CRP (mg/dL), median (IQR)	1.2 (0.4–6.2)	1.1 (0.5–4.3)	0.9 (0.3–8.8)
Β12 (ng/mL), median (IQR)	230 (168–329)	206 (151–339.5)	258 (195–296)
Albuminuria, *n* (%)	192 (43.9)	139 (52.1)	53 (31.2)
Nitrites, *n* (%)	22 (5)	19 (7.1)	3 (1.8)
Leucocyte esterase, *n* (%)	59 (13.5)	45 (16.9)	14 (8.2)

T2D: type 2 diabetes ASB: asymptomatic bacteriuria; SD: standard deviation; BMI: body mass index; DM: diabetes mellitus; IQR: interquartile range; HbA1c: glycated hemoglobin; UTI: urinary tract infection; UTIs: urinary tract infections; CRP: C-reactive protein; B12: vitamin B12.

**Table 2:** Characteristics of male and female patients with T2D and ASB.

	Patients with T2D and ASB (*n* = 88)	Patients with T2D without ASB (*n* = 349)	*p*
Age (years), mean (SD)	75.6 (9.8)	69.1 (9.1)	<0.0001
BMI (kg/m^2^), mean (SD)	33 (5)	29.4 (5.7)	<0.0001
Duration of T2D (years), median (IQR)	18 (10–22)	10 (6–15)	<0.0001
Family history of T2D, *n* (%)	58 (65.9)	220 (63)	0.7101
HbA1c, mean (SD)	7.4 (1)	6.7 (0.8)	<0.0001
Prior antimicrobial use, *n* (%)	13 (14.8)	16 (4.6)	0.0027
UTI within one year, *n* (%)	49 (55.7)	29 (8.3)	<0.0001
Recurrent UTIs, *n* (%)	10 (11.4)	2 (0.6)	<0.0001
Recent sexual contact, *n* (%)	15 (17)	15 (4.3)	0.0001
CRP (mg/dL), median (IQR)	1 (0.5–3)	1.3 (0.3–9)	0.6795
Β12 (ng/mL), median (IQR)	180 (143–256)	294.5 (233.5–373)	<0.0001
Albuminuria, *n* (%)	65 (73.9)	127 (36.4)	<0.0001
Nitrites, *n* (%)	19 (21.6)	3 (0.9)	<0.0001
Leucocyte esterase, *n* (%)	54 (61.4)	5 (1.4)	<0.0001

SD: standard deviation; BMI: body mass index; T2D: type 2 diabetes mellitus; IQR: interquartile range; HbA1c: glycated hemoglobin; UTI: urinary tract infection; UTIs: urinary tract infections; CRP: C-reactive protein; B12: vitamin B12.

**Table 3:** Characteristics of male and female patients with T2D and ASB.

	Male Diabetic Patients with ASB (*n* = 16)	Female Diabetic Patients with ASB (*n* = 72)	*p*
Age (years), mean (SD)	73.8 (9.7)	76 (9.9)	0.4294
BMI (kg/m^2^), mean (SD)	30.7 (4.2)	33.5 (5.1)	0.0455
Duration of DM (years), median (IQR)	15.5 (10–18.8)	18 (12–24.3)	0.2277
Family history of DM, *n* (%)	7 (43.8)	51 (70.8)	0.0468
HbA1c, mean (SD)	7.7 (1.3)	7.4 (0.9)	0.2891
Prior antimicrobial use, *n* (%)	4 (25)	9 (12.5)	0.2431
UTI within one year, *n* (%)	6 (37.5)	43 (59.7)	0.1633
Recurrent UTIs, *n* (%)	2 (12.5)	8 (11.1)	1
Recent sexual contact, *n* (%)	3 (18.8)	12 (16.7)	1
CRP (mg/dL), median (IQR)	4 (4.5)	2.7 (4.2)	0.1405
Β12 (ng/mL), median (IQR)	217 (154–309)	178 (141.3–247)	0.2821
Albuminuria, *n* (%)	14 (87.5)	51 (70.8)	0.2199
Nitrites, *n* (%)	3 (18.8)	16 (22.2)	1
Leucocyte esterase, *n* (%)	12 (75)	42 (58.3)	0.2656

SD: standard deviation; BMI: body mass index; DM: diabetes mellitus; IQR: interquartile range; HbA1c: glycated hemoglobin; UTI: urinary tract infection; UTIs: urinary tract infections; CRP: C-reactive protein; B12: vitamin B12.

**Table 4:** Microbiology of ASB in patients with T2D.

Species	All Patients, *n* (%)	Female Patients, *n* (%)	Male Patients, *n* (%)
*Escherichia coli*	55 (62.5)	50 (69.4)	5 (31.3)
*Streptococcus agalactiae*	9 (10.2)	7 (9.7)	2 (12.5)
*Klebsiella pneumoniae*	6 (6.8)	5 (6.9)	1 (6.3)
*Enterococcus faecalis*	6 (6.8)	2 (2.8)	4 (25)
*Proteus mirabilis*	6 (6.8)	5 (6.9)	1 (6.3)
*Pseudomonas aeruginosa*	2 (2.7)	1 (1.4)	1 (6.3)
*Staphylococcus haemolyticus*	1 (1.1)	1 (1.4)	0 (0)
*Acinetobacter lwoffi*	1 (1.1)	0 (0)	1 (6.3)
*Providencia stuartii*	1 (1.1)	0 (0)	1 (6.3)
*Streptococcus bovis*	1 (1.1)	1 (1.4)	0 (0)

**Table 5:** Logistic regression analysis of T2D patients presenting with ASB.

Variable	Univariate Analysis *p*	Multivariate Analysis *p*	Multivariate Analysis OR (95% CI)
Female gender	<0.001	0.271	2.216 (0.538–9.135)
Age	<0.001	0.169	1.062 (0.975–1.157)
BMI	<0.001	0.275	1.106 (0.923–1.324)
Duration of DM	<0.001	0.178	1.072 (0.969–1.187)
Hypertension	0.092		
Heart disease	0.0736		
PVD	0.3028		
Lung disease	0.5086		
CVD	0.0886		
Dyslipidemia	0.0982		
Renal disease	0.4444		
Renal failure	0.0937		
WBC count	0.2122		
Platelets	0.9359		
ESR	0.1669		
HbA1c	<0.001	0.005	3.921 (1.521–10.109)
Glucose	0.6565		
Creatinine	0.5968		
Albuminuria	0.9666		
UTI in previous year	<0.001	0.004	13.254 (2.245–78.241)
Β12 (per ng/mL)	<0.001	0.018	0.994 (0.989–0.999)

OR: odds ratio; CI: confidence interval; BMI: body mass index; DM: diabetes mellitus; PVD: peripheral vascular disease; CVD: cerebrovascular disease; WBC: white blood cells; ESR: erythrocyte sedimentation rate; HbA1c: glycated hemoglobin; UTI: urinary tract infection; B12: vitamin B12.

## Data Availability

The data presented in this study are available on request from the corresponding authors.

## Supplementary Materials

The following supporting information can be downloaded at: https://www.mdpi.com/article/10.3390/idr15010005/s1, Table S1. Characteristics of type 2 diabetic (T2D) female patients with and without asymptomatic bacteriuria (ASB); Table S2. Characteristics of T2D male patients with and without ASB.
