# Supplementary material for: Asymptomatic Bacteriuria in Patients with Type 2 Diabetes Mellitus
Source: Infect Dis Rep. 2023 Jan 5;15(1):43–54. doi: 10.3390/idr15010005 (PMC9887587; doi:10.3390/idr15010005)
Supplement: Supplementary file 1 [file idr-15-00005-s001.zip › idr-2109473-supplementary.pdf]

Table S1. Characteristics of type 2 diabetic (T2D) female patients with and without asymptomatic bacteriuria (ASB).

	**All female patients with T2D (*n*=267)**	**Female patients with T2D and ASB (*n*=72)**	**Female patients with T2D without ASB (*n*=195)**	**p**
**Age (years), mean (SD)**	70.6 (9.7)	76 (9.9)	68.7 (8.9)	<0.0001
**BMI (kg/m^2), mean (SD)**	31.5 (6)	33.5 (5.1)	30.7 (6.1)	0.0006
**Duration of DM (years), median (IQR)**	12 (7-19)	18 (12-24.3)	10 (6-15)	<0.0001
**Family history of DM, *n* (%)**	176 (65.9)	51 (70.8)	125 (64.1)	0.3828
**HbA1c, %, mean (SD)**	6.9 (0.9)	7.4 (0.9)	6.7 (0.8)	<0.0001
**Prior antimicrobial use, *n* (%)**	15 (5.6)	9 (12.5)	6 (3.1)	0.0058
**UTI within one year, *n* (%)**	68 (25.5)	43 (59.7)	25 (12.8)	<0.0001
**Recurrent UTIs, *n* (%)**	9 (3.4)	8 (11.1)	1 (0.5)	0.0001
**Recent sexual contact, *n* (%)**	18 (6.7)	12 (16.7)	6 (3.1)	0.0003
**CRP (mg/dl), median (IQR)**	1.1 (0.5-4.3)	1 (0.4-2.7)	1.5 (0.7-8)	0.1154
**B12 (ng/ml), median (IQR)**	206 (151-339.5)	178 (141.3-247)	335 (298-565.8)	<0.0001
**Albuminuria, *n* (%)**	139 (52.1)	51 (70.8)	88 (45.1)	0.0002
**Nitrites, *n* (%)**	19 (7.1)	16 (22.2)	3 (1.5)	<0.0001
**Leucocyte esterase, *n* (%)**	45 (16.9)	42 (58.3)	3 (1.5)	<0.0001

ASB: asymptomatic bacteriuria; SD: standard deviation; BMI: body mass index; DM: diabetes mellitus; IQR: interquartile range; HbA1c: glycated hemoglobin; UTI: urinary tract infection; UTIs: urinary tract infections; CRP: C-reactive protein; B12: vitamin B12.

Table S2. Characteristics of T2D male patients with and without ASB.

	All male patients with T2D (*n*=170)	Male patients with T2D and ASB (*n*=16)	Male patients with T2D without ASB (*n*=154)	p
**Age (years), mean (SD)**	70.0 (9.6)	73.8 (9.7)	69.6 (9.5)	0.0926
**BMI (kg/m²), mean (SD)**	28.1 (4.7)	30.7 (4.2)	27.8 (4.7)	0.019
**Duration of DM (years), median (IQR)**	12 (7-16)	15.5 (10-18.8)	12 (6-16)	0.097
**Family history of DM, *n* (%)**	102 (60)	7 (43.8)	95 (61.7)	0.1867
**HbA1c, mean (SD)**	6.8 (1)	7.7 (1.3)	6.7 (1)	0.0001
**Prior antimicrobial use, *n* (%)**	14 (8.2)	4 (25)	10 (6.5)	0.0297
**UTI within one year, *n* (%)**	10 (5.9)	6 (37.5)	4 (2.6)	<0.0001
**Recurrent UTIs, *n* (%)**	3 (1.8)	2 (12.5)	1 (0.6)	0.0237
**Recent sexual contact, *n* (%)**	12 (7.1)	3 (18.8)	9 (5.8)	0.0891
**CRP (mg/dl), median (IQR)**	0.9 (0.3-8.8)	2.4 (0.9-7.5)	0.8 (0.2-9.4)	0.3088
**B12 (ng/ml), median (IQR)**	258 (195-296)	217 (154-309)	266.5 (199.5-299.5)	0.2059
**Albuminuria, *n* (%)**	53 (31.2)	14 (87.5)	39 (25.3)	<0.0001
**Nitrites, *n* (%)**	3 (1.8)	3 (18.8)	0 (0)	0.0007
**Leucocyte esterase, *n* (%)**	14 (8.2)	12 (75)	2 (1.3)	<0.0001

ASB: asymptomatic bacteriuria; SD: standard deviation; BMI: body mass index; DM: diabetes mellitus; IQR: interquartile range; HbA1c: glycated hemoglobin; UTI: urinary tract infection; UTIs: urinary tract infections; CRP: C-reactive protein; B12: vitamin B12.
